# Supplementary material for: Effect of Injection Overmolding Parameters on the Interface Bonding Strength of Hybrid Thermoset–Thermoplastic Composites
Source: Polymers (Basel). 2023 Jun 29;15(13):2879. doi: 10.3390/polym15132879 (PMC10347229; doi:10.3390/polym15132879)
Supplement: Supplementary file 1 [file polymers-15-02879-s001.zip › polymers-2436572-supplementary.pdf]

Supplemental data

Figure S1 shows the schematic diagram of the lap shear test of overmolded hybrid thermoset-thermoplastic sample. The sample is placed on the fixture as shown in the figure.

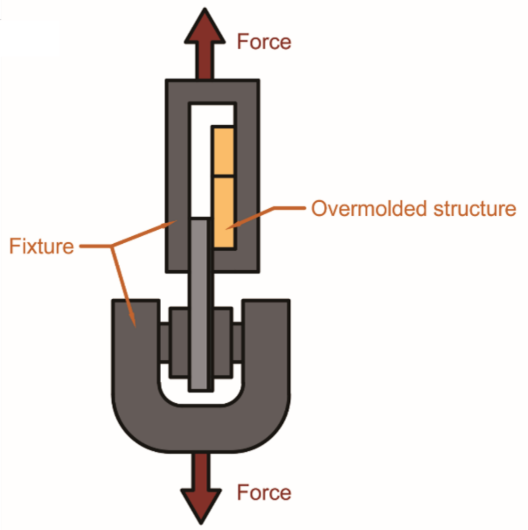

FIGURE S1. Schematic diagram of lap shear tests for overmolded hybrid thermoset-thermoplastic sample.
